# p53 and its isoforms in cancer

**DOI:** 10.1038/sj.bjc.6603886

**Published:** 2007-07-17

**Authors:** J-C Bourdon

**Affiliations:** 1 Inserm European Associated Laboratory, Department of Surgery and Molecular Oncology, University of Dundee, Inserm U858, CR-UK Cell Transformation Research Group, Dundee, UK

**Keywords:** splice, promoter, tumour, transcription, apoptosis, cell cycle

## Abstract

p53, p63 and p73 are members of the p53 gene family involved in development, differentiation and response to cellular stress. p53 gene is a transcription factor essential for the prevention of cancer formation. The p53 pathway is ubiquitously lost in human cancer either by p53 gene mutation (60% of cancers) or by lost of cell signalling upstream and downstream of p53 in the remaining cancers expressing WTp53 gene. As p53 pathway inactivation is a common denominator to all cancers, the understanding of p53 tumour suppressor activity is likely to bring us closer to cancer therapy. However, despite all the experimental evidences showing the importance of p53 in preventing carcinogenesis, it is difficult in clinical studies to link p53 status to cancer treatment and clinical outcome. The recent discovery that p53 gene encodes for nine different p53 proteins (isoforms) may have a profound impact on our understanding of p53 tumour suppressor activity. Studies in several tumour types have shown that the nine different p53 isoforms are abnormally expressed in tumour tissues compared to normal cells. p53 protein isoforms modulate p53 transcriptional activity and cell fate outcome in response to stress. Regulation of p53 function in normal and tumour tissues in man is likely to be more complex than has been hitherto appreciated. Therefore, the tumour p53 status needs to be determined more accurately by integrating p53 isoform expression, functional p53 mutation analysis and a panel of antibodies specific of p53 and of its target genes.

The response to cellular damage is complex involving the recognition of the damages and repair of the lesions, in particular, DNA damages to minimise the risk of genetic instability. Therefore, mutations or alterations of protein expression involved in this process predispose to genome instability, cancer and other pathologies (Kops *et al*, 2005). A central player in protecting the integrity of the genome is p53. The importance of its role is exemplified by the facts that p53 activity is ubiquitously lost in human cancer either by p53 protein inactivation or by *p53* gene mutation (for a review, see Iwakuma and Lozano, 2007; Lozano, 2007; Petitjean *et al*, 2007a; Vousden and Lane, 2007).

p53 protein is expressed at low levels under unperturbed conditions. However, the p53 pathway is activated by any cellular stresses that alter the normal cell-cycle progression or can induce mutations of the genome leading to the transformation of a normal cell into a cancerous cell. Depending on the tissue-type and the extend of the damage, activated p53 protein either stops the cell cycle to repair the lesions or switches ‘on’ the programmed cell death pathways (apoptosis), forcing the damaged cells to ‘commit suicide’. The p53 protein prevents the multiplication of damaged cells that are more likely to contain mutations and exhibit abnormal cellular growth than undamaged cells. Hence, p53 protein is the guardian of the genome preventing cancer formation (Lane, 1992).

The mechanisms by which p53 accomplishes its tumour suppressor activity are still not completely understood. The best-described mechanism is its ability to modulate gene expression. p53 is a transcription factor that binds directly and specifically as a tetramer to target sequences of DNA (p53-responsive elements (p53RE)) (El-Deiry *et al*, 1992; Funk *et al*, 1992; Bourdon *et al*, 1997). The ability of p53 to modulate gene expression is required for its tumour suppressor activity. Identification of the cyclin-dependent kinase inhibitor Waf as a p53-responsive gene, helps to explain how p53 can induce cell-cycle arrest (El-Deiry *et al*, 1993; Harper *et al*, 1993). Recently, several p53-inducible genes that encode for proteins with apoptotic potential have been identified (Chipuk and Green, 2006). However, the tumour suppressor p53 can trigger cell death independently of its transcriptional activity through subcellular translocation and activation of proapoptotic Bcl-2 family members (Moll *et al*, 2005).

## HUMAN P63 AND P73 ISOFORMS

Two *p53*-related genes, *p63* and *p73*, were identified in 1997 (Kaghad *et al*, 1997; Yang *et al*, 1998). The high level of sequence similarity in the DNA-binding domain between p53 protein family members allows p63 and p73 to transactivate p53-responsive genes causing cell-cycle arrest and apoptosis. However, p53, p63 and p73 proteins are not entirely functionally redundant as each p53-family transgenic knockout mice develop distinct phenotypes, illustrating that *p53*, *p63* and *p73* have specific biological functions (for a review, see Murray-Zmijewski *et al*, 2006).

We recently published that the *p53* gene family (p53/p63/p73) has a dual gene structure conserved in *Drosophila*, zebrafish and man (Bourdon *et al*, 2005; Chen *et al*, 2005). Like most of the genes in the human genome (Stamm *et al*, 2005), *p53* gene family members express multiple mRNA variants due to multiple splicing and alternative promoters. Hence, *p53* gene family members express different forms of p53 protein containing different domain of the protein (isoforms).

**Figure 1:**
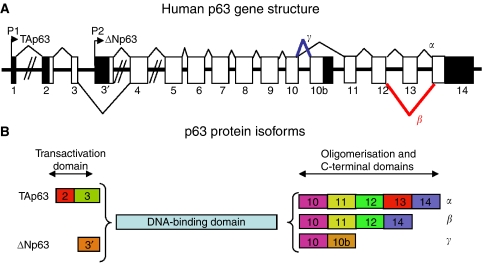
Human p63. (**A**) Schema of the human p63 gene structure: alternative splicing (*α*, *β*, *γ*) and alternative promoters (P1 and P2) are indicated. (**B**) p63 protein isoforms: TAp63 proteins encoded from promoter P1 contain the conserved N-terminal domain (Fxx*ψ*W) of transactivation (TA). ΔNp63 proteins encoded from promoter P2 are amino-truncated proteins containing an N-terminal domain different from TAp63 proteins. Numbers indicate the exons encoding p63 protein isoforms.

The human and mouse *p63* genes express at least three alternatively spliced C-terminal isoforms (*α*, *β*, *γ*) and can be transcribed from an alternative promoter located in the intron 3 (Figure 1A). The transactivating isoforms (TAp63) are generated by the promoter upstream of exon-1, whereas the alternative promoter in intron 3 leads to the expression of N-terminal-truncated p63 isoforms (ΔNp63) containing a different N-terminal domain. Altogether, the *p63* gene expresses at least six mRNA variants which encode for six different p63 protein isoforms (TAp63*α*, TAp63*β*, TAp63*γ*, ΔNp63*α*, ΔNp63*β* and ΔNp63*γ*). Although ΔNp63 isoforms lack the transactivation domain present in TAp63 isoform, they can transactivate through a different transactivation domain present in their distinct N-terminal end (Helton *et al*, 2006) (Figure 1B). p63 isoforms are able to bind to DNA through p53RE and p63RE to regulate transcription of target genes involved in differentiation, cell-cycle arrest or apoptosis (Murray-Zmijewski *et al*, 2006; Stiewe, 2007). Genetic experiments on mice have shown that p63 is essential for epidermal morphogenesis and limb development. p63-null animals do not survive beyond a few days after birth, show craniofacial malformations, limb truncations and fail to develop skin and other epithelial tissues (Mills *et al*, 1999).

**Figure 2:**
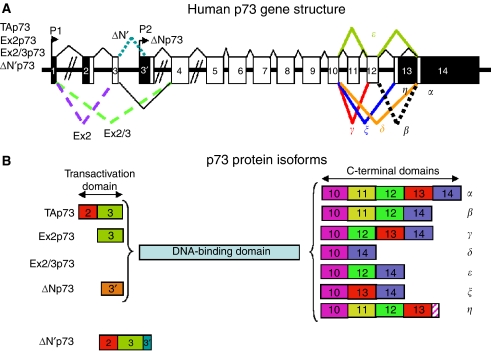
Human p73. (**A**) Schema of the human p73 gene structure: alternative splicing (*α*, *β*, *γ*, *ζ*, Δ, *ε*, *η*) and alternative promoters (P1 and P2) are indicated. (**B**) p73 protein isoforms: TAp73 proteins encoded from promoter P1 contain the conserved N-terminal domain (Fxx*ψ*W) of transactivation (TA). Ex2p73 proteins are due to alternative splicing of exon 2. They have lost the conserved N-terminal domain (Fxx*ψ*W) of transactivation (TA), but still contain part of the transactivation domain (Exon-3). Ex2/3p73 proteins are due to alternative splicing of exons 2 and 3. They have entirely lost the TA domain and are initiated from exon 4. To our knowledge, the protein encoded by ΔN'p73 mRNA has not been described. ΔN'p73 variant is often overexpressed at the mRNA level in tumours. ΔN'p73 is due to alternative splicing of exon 3′ contained in intron 3. Theoretically, ΔN'p73 mRNA would encode either for a short p73 protein or p73 protein isoforms identical to ΔNp73. ΔN'p73 mRNA contains the normal initiation site of translation in exon 2 (ATG in perfect kozak sequence) and a stop codon in exon-3′. Therefore, it could encode for a short p73 protein composed only of the transactivation domain (Fxx*ψ*W). It is possible that translation of ΔN'p73 mRNA is initiated from the third ATG available present in exon 3′and leading to p73 protein identical to ΔNp73 protein isoforms. ΔNp73 proteins encoded from promoter P2 are amino-truncated proteins containing an N-terminal domain different from TAp73 proteins. Numbers indicate the exons encoding p73 protein isoforms.

Like *p63*, the *p73* gene can be transcribed from an alternative promoter located in the intron 3 (Figure 2A). The *p73* gene expresses at least seven alternatively spliced C-terminal isoforms (*α*, *β*, *γ*, Δ, *ε*, *ζ*, *η*) and at least four alternatively spliced N-terminal isoforms, which contain different parts of the transactivation domain. Altogether, the *p73* gene expresses at least 35 mRNA variants, which can encode theoretically 29 different p73 protein isoforms (Figure 2B). p73 isoforms encoded by alternatively spliced exon2 and/or exon-3 mRNA variants are initiated at different ATG and contain therefore different part of the N-terminal domain, suggesting that they can have distinct protein interactions and specific activities. p73 isoforms are able to bind specically to DNA through p53RE and p73RE and activate transcription of target genes. Like p53, such activation can induce cell-cycle arrest or apoptosis (Murray-Zmijewski *et al*, 2006; Stiewe, 2007). Mice, functionally decient for all p73 isoforms, exhibited profound defects, including hippocampal dysgenesis, hydro–cephalus, chronic infections and inammation, as well as abnormalities in pheromone sensory pathways; however, did not show any increase susceptibility to cancer (Yang *et al*, 2000).

## HUMAN P53 ISOFORMS

**Figure 3:**
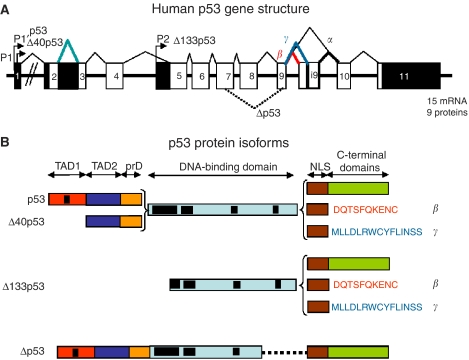
Human p53. (**A**) Schema of the human p53 gene structure: Alternative splicing (*α*, *β*, *γ*) and alternative promoters (P1, P1′ and P2) are indicated. p53 protein isoforms: (**B**) p53, p53*β* and p53*γ* proteins encoded from P1 or P1′ promoters contain the conserved N-terminal domain (Fxx*ψ*W) of transactivation (TA). Δ133p53 isoforms encoded from promoter P2 are amino-truncated proteins deleted of the entire TA domain and deleted of part of the DNA-binding domain. Translation is initiated at ATG-133. Δ40p53 protein isoforms encoded from P1 or P1′ promoters are amino-truncated proteins due to alternative splicing of exon 2 and/or alternative initiation of translation at ATG-40). Δ40p53 protein isoform have lost the conserved N-terminal domain of transactivation (Fxx*ψ*W), but still contain part of the transactivation domain. Δp53 protein isoform is due to noncanonical alternative splicing between the exon 7 and 9. Δp53 has lost 66 residues including the highly conserved domain V of the DNA-binding domain. The isoforms Δp53*β*, Δp53*γ*, Δ40Δp53, Δ40Δp53*β*, Δ40Δp53*γ*, Δ133Δp53, Δ133Δp53*β* and Δ133Δp53*γ* should theoretically be generated.

We recently established that human *p53* gene has indeed a dual gene structure similar to *p73* and *p63* genes (Bourdon *et al*, 2005). *p53* gene transcription can be initiated in normal human tissue from two distinct sites upstream of exon1 and from an internal promoter located in intron 4 (Figure 3A). The alternative promoter leads to the expression of an N-terminally truncated p53 protein initiated at codon 133 (Δ133p53). The intron 9 can be alternatively spliced to produce three isoforms: p53, p53*β* (identical to p53i9) and p53*γ*, where the p53*β* and p53*γ* isoforms lack the oligomerisation domain. Therefore, the human *p53* gene can encode at least nine different p53 protein isoforms, p53, p53*β*, p53*γ*, Δ133p53, Δ133p53*β* and Δ133p53*γ* due to alternative splicing of the intron 9 and usage of the alternative promoter in intron 4, and also Δ40p53, Δ40p53*β*, Δ40p53*γ* due to alternative splicing of the intron 9 and alternative initiation of translation or alternative splicing of the intron 2 (Ghosh *et al*, 2004) (Figure 3). p53 variant mRNA are expressed in several normal human tissues in a tissue-dependent manner, indicating the internal promoter and the alternative splicing of *p53* can be regulated. Moreover, the tissue-specific expression of the p53 isoforms could explain the tissue-specific regulation of p53 transcriptional activity in responses to stresses such as ionising radiation, UV, pH and hypoxia. Thus, the liver induces *p21* and cell-cycle arrest in a p53-dependent manner, whereas the spleen and thymus induce a massive p53-dependent apoptosis in response to the same dose of ionising radiation. (Midgley *et al*, 1995; Bouvard *et al*, 2000; Fei *et al*, 2002).

We have shown using commonly available p53 antibodies that endogenous p53 isoforms are expressed at the protein level. However, such antibodies cannot identify specifically the p53 isoforms. It is only by raising a specific anti-p53*β* antibody that we demonstrated expression of the endogenous p53*β* and Δ133p53*β* protein isoforms. This implies that p53*γ*, Δ133p53*γ* and Δ133p53 are expressed at the protein level. P53 isoforms have different subcellular localisations, suggesting that they can have distinct activities. Like full-length p53, Δ133p53 and p53*β* are localised mainly in the nucleus with a minor staining in the cytoplasm. In most cells, p53*γ* is localised in the nucleus, but can be localised in the cytoplasm in some cells, suggesting that p53*γ* could be shuttling between the nucleus and the cytoplasm. In most cells, Δ133p53*β* protein is localised in the nucleus and the cytoplasm, but can form in 10% of cells foci in the nucleus that are different from nucleoli. Contrary to Δ133p53*β*, Δ133p53*γ* is localised only in the cytoplasm, indicating that the C-terminal amino acids (*β* and *γ*) can modify the subcellular localisation of these isoforms (Bourdon *et al*, 2005).

Interestingly, the dual gene structure of the *p53* gene is conserved in human, *Drosophila* (Bourdon *et al*, 2005), mouse (Bourdon, unpublished data) and zebrafish (Chen *et al*, 2005), whereas the alternative splicing is species-specific. It suggests an unforeseen complex regulation that may play a major role in controlling p53 activity. Like p63 or p73 isoforms, p53 isoforms can have distinct biochemical activities.

p53*β* binds preferentially the p53-responsive promoters p21 and Bax rather than Mdm2, whereas p53 binds preferentially to Mdm2 and p21 rather than Bax promoters. p53*β* can form a protein complex with p53 and can specifically enhance p53 transcriptional activity at the Bax promoter in response to cellular stress, whereas it has no effect on p53 activity in absence of stress (Bourdon *et al*, 2005). Co-transfection of p53 with p53*β* increases slightly p53-mediated apoptosis, whereas co-transfection of p53 with Δ133p53 strongly inhibits p53-mediated apoptosis in a dose-dependent manner. This indicates that wild-type p53 activity may be modulated in the presence of p53 isoforms, and thus that regulation of p53 function in normal and tumour tissues in human is likely to be more complex than has been hitherto appreciated. Moreover, each p53 protein isoform may have specific biological activities independent of full-length p53. This may explain how p53 can be involved in the regulation of so many biological functions (i.e. cell-cycle arrest, apoptosis, differentiation, replication, DNA repair, meiosis, mitosis, etc…).

Deregulation of p53 isoforms expression may play a role early in tumour formation, as attenuation of the WT p53 response would render the cells more susceptible to further genetic damage and therefore to neoplastic transformation and tumour progression. Tumours with abnormal p53 isoform expression would have a predicted phenotype of WT p53 by sequence but compromised p53 activity. Such hypothesis is consistent with experimental evidences showing abnormal expression of p53 isoforms in head and neck, acute myeloid leukaemia (AML) and breast tumours (Bourdon *et al*, 2005; Anensen *et al*, 2006; Boldrup *et al*, 2007). Whereas only 25% of breast tumours express mutant p53, p53*β* and p53*γ* expressions are frequently lost (60%) and Δ133p53 is frequently overexpressed in breast tumours. The abnormal p53 isoforms expression can therefore account for loss of p53 tumour suppressor activity in breast tumour. In AML, where *p53* gene is mutated only in 10% of the cases, abnormal p53 isoform expression at the mRNA and protein levels has been reported (Anensen *et al*, 2006). The authors studied p53 isoform expression after chemotherapy in patients using RT-PCR and two-dimensional immunoblot. They reported a rapid shift within 4 h after chemotherapy from a shorter p53 protein isoform toward full-length p53 protein expression in AML patient, indicating that expression of p53 protein isoform could be modulated *in vivo* in response to chemotherapy.

This strongly suggests that the differential expression of p53 isoforms could disrupt the p53 response and contribute to tumour formation (Anensen *et al*, 2006). Moreover, it may provide some explanation to the difficulties in many clinical studies to link p53 status to the biological properties and drug sensitivity of human cancers as p53 status is determined either by sequencing and/or immunohistochemistry.

## ΔP53 ISOFORMS

It has recently been reported the intra-exons splicing of the exons 7 to 9 of *p53*, which leads to a p53 isoform deleted of the conserved box V in the DNA-binding domain (Δp53) (Rohaly *et al*, 2005). This noncanonical splicing is not consistent with any known rules of splicing, which are strictly conserved through eukaryotes (from yeast to human) (Stamm *et al*, 2005). Δp53 mRNA was isolated after reverse transcription at 37°C using MMLV reverse transcriptase. Based on our experience, such conditions give rise to cDNA with noncanonical intra-exons splicing after RT-PCR amplification probably because mRNA stem–loop structures are not destroyed at 37°C. As we perform reverse transcription at 45°C using AMV reverse transcriptase, we have never amplified cDNA with noncanonical intra-exons splicing. Despite all our efforts, we could not detect by RT-PCR, the mRNAs of Δp53 or of its theoretical spliced variant Δp53*β*, Δp53*γ*, Δ133Δp53, Δ133Δp53*β* and Δ133Δp53*γ* in 21 normal human tissue analysed nor in numerous tumours of several human tissue origins. Several studies have already confirmed detection and abnormal expression of p53*β*, p53*γ*, Δ133p53, Δ133p53*β* and Δ133p53*γ* in head and neck tumours (Boldrup *et al*, 2007), AML (Anensen *et al*, 2006) and in cell lines (Goldschneider *et al*, 2006). However, none of those studies reported detection of the Δp53 mRNA, although the primers used would have allowed its amplification by PCR.

Then, Rohaly *et al* (2005), attempt to demonstrate endogenous Δp53 protein expression using a panel of p53 monoclonal antibodies, DO-1 (epitope 21–25 aa), ICA-9 (epitope 383–393 aa) and DO-12 whose epitope (256–270 aa) is lost in Δp53 protein. The author could detect a p53 band at 45 kDa with DO-1 and ICA-9 antibodies but not with DO-12 antibody, which was interpreted as proving the expression of the Δp53 isoform. However, this should be interpreted with cautious, as we showed that p53*β*, p53*γ* and Δ40p53 migrate all at 45 kDa (Bourdon *et al*, 2005). In our hand, we can detect p53*β*, p53*γ* and Δ40p53 at 45 kDa with DO-12 antibody after long exposure. It is thus likely that Rohaly *et al*'s (2005) experimental conditions were not optimised to detect the 45 kDa band (i.e. short exposure, high antibody dilution,…). Therefore, the 45 kDa band identified by Rohaly *et al* (2005) is probably composed of a mix of Δp53, p53*β*, p53*γ* and Δ40p53.

To further confirm that the 45 kDa band is Δp53 isoform, Rohaly *et al* (2005) immunoprecipitated the 45 kDa band using p53 antibody and analysed it by MALTI-TOF/MS mass spectrometry. The masses of the tryptic fragments obtained from MALTI-TOF/MS were compared to the predicted p53 tryptic peptide map by peptide mass fingerprinting using database-matching procedure. The corresponding tryptic map assembled after MALDI-TOF/MS covered 88% of the p53 protein sequence, confirming that the 45 kDa band is composed of p53 protein. The same tryptic fragments that did not match full-length p53, are encountered in the predicted Δp53 tryptic peptide map, but are also encountered in a mix of the predicted p53*β* and Δ40p53 tryptic peptide maps. As the authors did not sequence the corresponding peptides, it is thus impossible to establish whether Δp53 is expressed. Only an antibody specific to Δp53 would prove unequivocally the endogenous expression of Δp53.

Δp53, which lacks a conserved domain of p53 in the DNA-binding domain, was reported to be transcriptionally active toward some p53 target genes and to be critical for the intra-S phase checkpoint (Rohaly *et al*, 2005). Contrary to previous publication, Chan and Poon (2007) recently reported with strong experimental evidences that Δp53 isoform lacks intrinsic transcriptional activity and lacks dominant-negative activity toward full-length p53. This is probably because Δp53 is not imported into the nucleus and stay in the cytoplasm. Therefore, further studies will be required to establish expression of the endogenous Δp53 isoforms and its biological relevance.

## DETERMINATION OF P53 STATUS BY SEQUENCING

The difficulty to link p53 mutation status to clinical outcome and cancer treatment can be explained by the fact that mutations of the *p53* gene do not necessarily result in inactivation of p53 transcriptional activity (Kato *et al*, 2003; Iacopetta *et al*, 2006; Petitjean *et al*, 2007b). Hence, 60% of the mutations that can occur in the *p53* gene do not alter p53 transcriptional activity. Only 15% of the mutations lead to mutant p53 completely inactive in transactivation. In the remaining 25%, mutants p53 present differential transcriptional activity. They can transactivate some promoters, but are completely inactive on others. It is therefore important to establish whether p53 mutation is correlated to loss of p53 function to determine accurately p53 status in clinical studies.

Moreover, p53 mutation analysis has to be re-evaluated in cancer in light of p53 isoform expression. p53 isoforms are encoded by exons different from full-length p53, mutations occuring upstream of codon 133 (exon-5) or downstream of codon 331 (exon-9), would affect some p53 isoforms, but not others. This may lead to the loss of some p53 biological activities, keeping others unaffected. Furthermore, although *p53* gene mutated at codon 133 substituting methionine to valine, leucine or isoleucine encodes for a p53 protein with similar transcriptional activity to wild-type p53, such a mutation prevents initiation of translation and thus expression of Δ133p53, Δ133p53*β* and Δ133p53*γ* isoforms (Kato *et al*, 2003; Iacopetta *et al*, 2006; Petitjean *et al*, 2007b). Similarly, mutations in introns of *p53* gene can alter p53 activity either by affecting alternative splicing or by altering the activity of the internal *p53* promoter located in intron 4. As p53 isoform can modulate p53 transcriptional activity and p53-mediated apoptosis, the ratio between p53 isoforms can be an important cell fate determinant. The changes upon stimuli of the balance and interactions between the isoforms are likely to be fundamental to our understanding in the transition between normal cell cycling and the onset of tumour formation.

## DETERMINATION OF P53 STATUS BY IMMUNOSTAINING

p53 immunostaining on tumour sections should be interpreted with cautious, as commonly available p53 antibodies can detect some p53 isoforms, but do not identify them specifically (Table 1). The mouse monoclonal antibodies DO-1 and DO-7 recognise p53, p53*β* and p53*γ*, but not the other p53 isoforms. The rabbit or sheep polyclonal p53 antibodies (CM1, SaPu, respectively) raised against recombinant full-length human p53 protein recognise all p53 isoforms, although Δ133p53*β* and Δ133p53*γ* are weakly revealed by polyclonal antibodies as these isoforms have lost most immunogenic domains of p53. The mouse monoclonal antibody, 1801, can reveal specifically most p53 isoforms except Δ133p53, Δ133p53*β* and Δ133p53*γ*. The mouse monoclonal antibody DO-12 has a weak affinity for p53 and requires a long exposure to reveal p53 isoforms. However, DO-12 is particularly efficient to immunoprecipitate Δ133p53, Δ133p53*β* and Δ133p53*γ* isoforms, which can be revealed by CM1 rabbit polyclonal antibody. The 421 mouse monoclonal antibody has also a low affinity for p53 mostly, because its epitope can be heavily modified by phosphorylations, ubiquitinylation, acetylation, methylation and neddylation. Although the 421 epitope is present in Δ40p53 and Δ133p53 isoforms, we have never been able to detect these isoforms using the 421 antibody. The rabbit polyclonal KJC8 antibody is specific of p53*β* isoforms and can be used in paraffin-embedded tissue. Novel specific p53 isoforms antibodies are being generated and will allow a clear identification of the p53 isoforms in the near future.

p53 isoforms have different subcellular localisations. Therefore, the use of a panel of p53 antibodies in immunohistochemistry can give different staining patterns for p53 in a same tumour sample. It would be interesting to investigate whether some p53 subcellular localisation can be associated with clinical markers. To have a better understanding of p53 immunostaining, it should be completed by RT-PCR analysis to determine p53 isoform mRNA expression in tumours.

Detection of p53 isoform expression by RT-PCR requires high-quality RNA. As RNAs are rapidly degraded in tissue in absence of blood supply (half-life of 15 min), RNAs have to be extracted immediately after biopsies or after surgical resection or on tumour samples nitrogen-frozen immediately after surgical resection. After purification, only total RNA extracts presenting a suitable ratio *28S/18S*⩾1.5 should be used for RT-PCR to determine p53 isoform expression in tumours. Such RNA quality is very difficult to obtain from paraffin-embedded tumour section as paraffin treatment destroys low abundant mRNA. Therefore, it is not recommended to use paraffin-embedded sections as source of RNA for RT-PCR of p53 isoform.

Therefore, determination of p53 status in clinical studies is much more complex than hitherto appreciated. It suggests that it requires an integrated and complex analysis of p53 isoform expressions associated with p53 mutation analysis and immunohistochemistry. To date, no clinical studies have integrated all those p53 parameters to determine p53 status. Several clinical studies attempted to establish p53 status by analysing p53 target gene expression either by immunostaining or RNA microarray. Unfortunately, there are no established gene or protein markers of p53 functional activity yet. However, one can expect that future studies will be able to identify some. It would simplify greatly the determination of p53 status in tumours and help to diagnosis and cancer treatment.

## Figures and Tables

**Table 1:** p53 isoform-specific antibodies

	**Mouse monoclonal**				**Rabbit polyclonal**	
**Epitope (aa)**	**DO-1/DO-7 (20–25)**	**1801 (46–55)**	**DO-12 (256–270)**	**421 (372–382)**	**KJC8 (*β*)**	**CM1 recomb p53**
p53	++	++	+	+	−	+++
p53*β*	++	++	+	−	++	++
p53*γ*	++	++	+	−	−	++
Δ40p53	−	++	+	−	−	++
Δ40p53*β*	−	++	+	−	++	++
Δ40p53*γ*	−	++	+	−	−	++
Δ133p53	−	−	+	−	−	+
Δ133p53*β*	−	−	++	−	++	+
Δ133p53*γ*	−	−	++	−	−	+
